# Association between socioeconomic status and metabolic control and diabetes complications: a cross-sectional nationwide study in Chinese adults with type 2 diabetes mellitus

**DOI:** 10.1186/s12933-016-0376-7

**Published:** 2016-04-05

**Authors:** Xiaoming Tao, Jihu Li, Xiaolin Zhu, Bin Zhao, Jiao Sun, Linong Ji, Dayi Hu, Changyu Pan, Yuxin Huang, Suyuan Jiang, Qiang Feng, Cuiping Jiang, on behalf of CCMR-3B STUDY Investigators

**Affiliations:** Department of Endocrinology, Huadong Hospital Affiliated to Fudan University, 221 yananxi road, Shanghai, 200040 China; MSD China Holding Co., Ltd., Shanghai, China; Department of Endocrinology and Metabolism, Peking University People’s Hospital, Beijing, China; Department of Cardiology, Peking University People’s Hospital, Beijing, China; Department of Endocrinology, The Chinese PLA General Hospital, Beijing, China

**Keywords:** Socioeconomic status, Type 2 diabetes, Blood glucose, Blood pressure, Blood lipids, Diabetes complications

## Abstract

**Background:**

Low socioeconomic status (SES) is associated with adverse cardiovascular risk factor patterns and poor outcomes in patients with diabetes. The aim of this study was to determine whether SES is associated with the control of blood glucose, blood pressure, blood cholesterol (3Bs), and diabetic complications in Chinese adults with type 2 diabetes.

**Methods:**

Data regarding patients’ demographics, social economics, diabetes complications, and cardiovascular risk profiles were analyzed for 25,454 patients. The outcomes of interest were the proportions of patients with HbA1c <7.0 %, blood pressure <140/80 mmHg, total serum cholesterol <4.5 mmol/L, and diabetes complications. Multivariable logistic regression was used for analysis.

**Results:**

Of the 25,454 patients, the least educated patients (1695, 6.7 %) had the highest chances of developing cardiovascular diseases (*p* = 0.048), cerebrovascular diseases (*p* < 0.001), and retinopathy (*p* < 0.001). The patients with lowest household income (10,039, 40.8 %) had the highest prevalence of retinopathy (*p* < 0.001) and neuropathy (*p* < 0.001). The most educated patients were more likely than the least educated patients to achieve HbA1c <7.0 % [adjusted odds ratio (OR) 1.38; 95 % confidence interval (95 % CI) 1.22–1.56] and 3B goals (adjusted OR 1.30; 95 % CI 1.11–1.53). The patients with highest household income were more likely to achieve BP < 140/80 mmHg (adjusted OR 1.16; 95 % CI 1.07–1.27), but less likely to reach HbA1c < 7.0 % (adjusted OR 0.90; 95 % CI 0.83–0.98) than those lowest income patients.

**Conclusions:**

Low SES was associated with poor metabolic control and more diabetes complications in adult patients in China. Individual diabetes management based on the SES of patients is encouraged.

**Electronic supplementary material:**

The online version of this article (doi:10.1186/s12933-016-0376-7) contains supplementary material, which is available to authorized users.

## Background

Patients with type 1 or type 2 diabetes who have a lower socioeconomic status (SES) experience worse clinical outcomes than those with a higher SES [1–7]. SES may influence multiply aspects of diabetes management including the quality of health care, availability of community resources, acquisition of diabetes-related knowledge, communication with providers, adherence to recommended medication, exercise intensity, and dietary regimens [8]. The prevalence of type 2 diabetes in adults has increased to 9.7 % in China, and only 27 % of Chinese patients with type 2 diabetes reach the target level of hemoglobin A1c (HbA1c) [9, 10]. Secrest et al. [11] have found that type 1 diabetes patients of lower SES may exhibit poorer self-management and thus experience more diabetes-related complications. During recent decades, education and income levels in China have been rapidly increasing with the development of economic and education reform. A large clinical study has revealed that the prevalence of diabetes in China is increasing with economic development, especially among individuals with lower levels of education and socioeconomic development [12]. However, few data are available to reveal whether the effects of SES are associated with the common risk factors of cardiovascular disease, such as diabetes control, hypertension, and dyslipidemia in Chinese patients with type 2 diabetes [13, 14]. Thus, the present study was undertaken to analyze the relationship between SES and metabolic control and diabetes complications in Chinese adults.

The Nationwide Assessment of Cardiovascular Risk Factors: Blood Glucose, Blood Pressure, and Blood Lipid in Chinese Patients with Type 2 Diabetes (3B STUDY) was designed and conducted under the guidance of the China Cardio metabolic Registries (CCMR) advisory board as a cohort study series with the aim to more fully describe the epidemiology and real-world clinical outcomes of cardiovascular and metabolic diseases in China [15].

## Methods

### Study population

The CCMR-3B STUDY was an observational, cross-sectional, multicenter, multispecialty study of outpatients with established type 2 diabetes who were diagnosed according to the World Health Organization criteria. The study design and population have been previously described [15, 16]. Patients were eligible if: (1) they aged 18 years or older, diagnosed with type 2 diabetes; (2) had a diabetes duration of at least 6 months at enrollment; and (3) were willing to sign a consent form and able to complete the questionnaires. Patients with type 1 diabetes and/or gestational diabetes were excluded from the study. A total of 25,454 patients were enrolled by 730 investigators between August 2010 and March 2011 at 104 hospitals across all major geographical regions in China. All of the 25,454 patients in the 3B STUDY were included in the present study as a subgroup, and they were stratified to four groups according to education level and to three groups according to household net income. The study was approved by the ethics committee of People’s Hospital Peking University and conducted in compliance with the principles in the Declaration of Helsinki. Informed consent was obtained from all participants and formally recorded.

### Data collection

Patient data were collected from medical charts and clinical examination records. SES and health behaviors (i.e., smoking status, alcohol consumption, and exercise intensity), medications, medication adherence, and medical histories were obtained by self-reporting during a face-to-face interview. Physical examination included anthropometry (weight, height, and waist circumference) and blood pressure measurements. Well-documented fasting serum glucose, total cholesterol (TC), low density lipoprotein-cholesterol (LDL-c), high density lipoprotein-cholesterol (HDL-c), and triglycerides (TG) measurements obtained within 1 month or measured at each study site during the outpatient visit were recorded. An HbA1c concentration known to have been obtained during the 3 months prior to the enrollment visit, or measured at enrollment, was recorded.

### Definition of variables

SES was measured according to self-reported education and income. The education categories were illiteracy, primary education, secondary education, and college and above. The categories of household net income were <¥2000, ¥2000–5000, and >¥5000 monthly. Diabetes was defined by self-reporting of a prior history of diabetes and/or current insulin or hypoglycemic medication use. Hypertension was defined as a documented diagnosis of hypertension and/or current use of anti-hypertensive agents. Dyslipidemia was defined according to previous diagnosis and/or use of lipid modifying therapy. Cardiovascular disease was defined as stable angina, unstable angina, myocardial infarction, and undergoing coronary bypass. Diabetic retinopathy, nephropathy, and neuropathy were recorded only if a well-documented diagnosis was available. Smoking was defined as consuming on average one cigarette per day for at least 1 year. A history of alcohol consumption was defined as having drunk on average 50 g alcohol per day for 1 year or longer. Sedentary behavior was evaluated according to the frequency and amount of time spent exercising. Sedentary lifestyle was defined as daily exercise less than 30 min. body mass index (BMI) was calculated as weight in kilograms divided by the square of height in meters (kg/m^2^).

### Clinical measures

Attainment of 3B study therapeutic control goals was evaluated for each patient. According to the China Guidelines for Type 2 Diabetes [17], attainment of glycemic goal was defined as an HbA1c <7 %, and the blood pressure goal was a systolic blood pressure (SBP) <140 mmHg and a diastolic blood pressure (DBP) <90 mmHg regardless of a history of hypertension or current antihypertensive treatment. The LDL-c goal was <2.6 mmol/l regardless of a history of dyslipidemia or current anti-hyperlipidemia treatment.

### Statistical methods

Statistical analyses were carried out using SAS 9.2 (SAS Institute Inc., Cary, NC, USA). Continuous data are expressed as mean ± standard deviation (SD) for data that were normally distributed and median (interquartile range) for data not normally distributed. Categorical data are presented in terms of absolute values and percentages. Continuous variables were analyzed using Student’s *t* test and analysis of variance (ANOVA). Categorical variables were analyzed using the Chi squared test and Fisher’s exact test. A multivariable logistic regression analysis was conducted to identify associations between dependent and independent variables. Variables with *p* < 0.2 in univariate analyses were entered into the multivariable phase. Only variables with *p* < 0.1 were retained in the final model after application of a backward elimination variable-selection procedure. The results were expressed as adjusted odds ratios (ORs) with 95 % confidence intervals (95 % CIs). All enrolled patients were identified by participant number in the database to ensure anonymity.

## Results

The characteristics of the patients stratified according to education attainment and household income are shown in Table 1. Over all, the age of the patients was 62.6 ± 11.89 years (mean ± SD), and 47.0 % were male. The total numbers of patients in the illiteracy, primary education, secondary education, and college and above groups were 1695 (6.7 %), 5667 (22.3 %), 11,936 (46.9 %), and 6156 (24.2 %), respectively. There were 10,039 (40.8 %), 11,586 (47.1 %), and 2964 (12.1 %) patients who had a household income <¥ 2000, ¥ 2000–5000 and >¥ 5000, respectively. The mean BMI was 24.8 ± 3.57 kg/m^2^, and the mean waist circumference was 83.5 ± 8.10 cm. Patients with higher education or income tended to be younger and male. In males, the most educated or highest income patients tended to weigh more. Male and female patients differed in smoking habits and alcohol consumption. Male patients with the highest income were more likely to currently smoke and drink; in contrast, in females, higher education was associated with a lower incidence of current smoking, and higher income was associated with reduced alcohol consumption. In both male and female patients, a higher level of education was inversely related to a sedentary lifestyle. Finally, patients with higher education and income presented better medication adherence.

**Table 1:** Characteristics of male or female patients illustrated by education and income levels

Variables	All patients	Education					Household net income^#^			
		Illiteracy	Primary education	Secondary education	College and above	*p*	<2000	2000–5000	≥5000	*p*
Male, n	11,955	277	1836	5738	4104		3941	5796	1804	
Age (y), mean ± SD	60.9 ± 12.65	70.9 ± 10.76	67.5 ± 11.28	60.3 ± 11.88	58.2 ± 13.04	<0.001	62.1 ± 11.90	61.1 ± 12.70	57.9 ± 13.53	<0.001
BMI (kg/m^2^), mean ± SD	24.8 ± 3.32	24.1 ± 3.36	24.4 ± 3.39	24.8 ± 3.36	25.1 ± 3.21	<0.001	24.5 ± 3.32	24.9 ± 3.30	25.2 ± 3.34	<0.001
WC (cm), mean ± SD	87.1 ± 7.75	86.2 ± 8.62	86.2 ± 8.07	87.1 ± 7.70	87.7 ± 7.55	<0.001	86.5 ± 7.73	87.4 ± 7.66	88.0 ± 7.67	<0.001
Diabetes duration (y), median (IQR)	5.8 (2.4-10.9)	5.8 (2.5–11.0)	5.7 (2.6–11.0)	5.8 (2.5–11.0)	5.8 (2.3–10.9)	0.316	5.5 (2.3–10.9)	5.9 (2.6–11.0)	5.8 (2.3–10.9)	0.011
Current smoker, n (%)	3888 (32.5)	74 (26.7)	499 (27.2)	2021 (35.2)	1294 (31.5)	0.007	1300 (33.0)	1805 (31.1)	647 (35.9)	0.001
Alcohol consumptions, n (%)	1944 (16.3)	41 (14.8)	254 (13.8)	935 (16.3)	714 (17.4)	0.007	588 (14.9)	953 (16.4)	353 (19.6)	0.001
Sedentary lifestyle, n (%)	4355 (36.4)	160 (57.8)	763 (41.6)	2100 (36.6)	1332 (32.5)	<0.001	1399 (35.5)	2139 (36.9)	683 (37.9)	0.104
Good medication adherence, n (%)	10,129 (84.7)	218 (78.7)	1541 (83.9)	4829 (84.2)	3541 (86.3)	<0.001	3250 (82.5)	4964 (85.6)	1536 (85.1)	<0.001
Female, n	13,499	1418	3831	6198	2025		6098	5790	1160	
Age (y), mean ± SD	64.0 ± 10.87	71.0 ± 9.34	67.2 ± 9.91	61.3 ± 10.19	61.1 ± 11.63	<0.001	64.2 ± 10.58	64.1 ± 10.83	62.4 ± 12.13	<0.001
BMI (kg/m^2^), mean ± SD	24.8 ± 3.78	24.7 ± 3.67	24.9 ± 3.93	24.9 ± 3.74	24.6 ± 3.68	0.024	24.8 ± 3.76	24.9 ± 3.83	24.6 ± 3.56	0.097
WC (cm), mean ± SD	78.9 ± 5.90	79.0 ± 6.07	79.0 ± 6.02	79.0 ± 5.71	78.4 ± 6.13	0.004	78.8 ± 5.93	79.0 ± 5.82	78.9 ± 5.90	<0.001
Diabetes duration (y), median (IQR)	6.8 (2.8–12.0)	7.1 (3.0–12.2)	7.2 (3.0-12.5)	6.6 (2.8–11.8)	6.7 (2.6–12.3)	<0.001	6.6 (2.7–11.5)	6.9 (2.9–12.6)	7.0 (2.9–12.9)	<0.001
Current smoker, n (%)	283 (2.1)	34 (2.4)	93 (2.4)	136 (2.2)	20 (1.0)	<0.001	145 (2.4)	105 (1.8)	22 (1.9)	<0.001
Alcohol consumptions, n (%)	67 (0.5)	10 (0.7)	17 (0.4)	29 (0.5)	11 (0.5)	0.318	31 (0.5)	28 (0.5)	5 (0.4)	0.306
Sedentary lifestyle, n(%)	4883 (36.2)	728 (51.3)	1591 (41.5)	1976 (31.9)	588 (28.7)	<0.001	2222 (36.4)	2064 (35.6)	438 (37.8)	0.013
Good medication adherence, n (%)	11,927 (88.4)	1175 (82.9)	3345 (87.3)	5533 (89.3)	1874 (91.3)	<0.001	5198 (85.2)	5258 (90.8)	1063 (91.6)	<0.001

414 male patients and 451 female patients without household income record. *BMI* body mass index; *WC* waist circumference

Continuous variables were analyzed using the Student’s *t* test and ANOVA. Categorical variables were analyzed using the Chi squared test and Fisher’s exact test

The pharmaceutical treatment patterns of patients divided by educational attainment and household income are shown in Table 2. A total of 13,988 patients (55.0 %) were treated with oral hypoglycemic drugs (OHDs), 4446 (17.5 %) with insulin, and 4620 (18.2 %) with both oral agents and insulin. Patients with higher education or income were more likely to use a combined treatment of OHD and insulin than those with less education or income. For patients treated with OHD alone, α-glucosidase inhibitor, thiazolidinediones (TZDs), and meglitinides were used more commonly in patients with more education than in those with less education, whereas biguanide, TZDs, and meglitinides were more frequently used in patients with a higher income than in patients with a lower income. The blood pressure lowering drugs used were beta-blockers in 2361 patients (9.3 %), calcium channel blockers in 6202 patients (24.4 %), angiotensin-converting enzyme (ACE) inhibitors in 2121 patients (8.3 %), and angiotensin II receptor antagonists in 4084 patients (16.0 %). Other medications included aspirin in 4693 patients (18.4 %) and statins in 5054 patients (19.9 %). The proportion of patients with a higher education level that was treated with beta-blockers, angiotensin II receptor antagonists, aspirin, and statins was greater than that of patients with a lower education level. Also, a greater proportion of patients with higher income used beta-blockers, angiotensin II receptor antagonists, and statins compared to patients with lower income.

**Table 2:** The pharmaceutical treatment patterns presented by education and income levels

Variables	All patients (n = 25,454)	Education				*p*	Household Net Income^a^			*p*
		Illiteracy (n = 1695)	Primary education (n = 5667)	Secondary education (11,936)	College and above (n = 6156)		<2000 (n = 10,039)	2000–5000 (n = 11,586)	≥5000 (n = 2964)	
OHD only, n (%)	13,988 (55.0)	988 (58.3)	3181 (56.1)	6564 (55.0)	3255 (52.9)	<0.001	5497 (54.8)	6525 (56.3)	1543 (52.1)	<0.001
Sulfonylureas, n (%)	6578 (47.0)	561 (56.8)	1670 (52.5)	3097 (47.2)	1250 (38.4)	<0.001	2732 (49.7)	3000 (46.0)	664 (43.0)	<0.001
Biguanide, n (%)	7623 (54.5)	497 (50.3)	1696 (53.3)	3662 (55.8)	1768 (54.3)	0.004	2930 (53.3)	3545 (54.3)	892 (57.8)	0.001
α-glucosidase inhibitor, n (%)	4432 (31.7)	266 (26.9)	897 (28.2)	2018 (30.7)	1251 (38.4)	<0.001	1422 (25.9)	2343 (35.9)	537 (34.8)	<0.001
Thiazolidinediones, n (%)	1378 (9.9)	70 (7.1)	298 (9.4)	654 (10.0)	356 (10.9)	0.003	450 (8.2)	656 (10.1)	217 (14.1)	<0.001
Meglitinides, n (%)	1146 (8.2)	55 (5.6)	234 (7.4)	528 (8.0)	329 (10.1)	<0.001	421 (7.7)	547 (8.4)	139 (9.0)	0.225
Insulin only, n ( %)	4446 ( 17.5)	300 (17.7)	1066 (18.8)	2035 (17.0)	1045 (17.0)	<0.001	1837 (18.3)	1881 (16.2)	495 (16.7)	<0.001
OHD + insulin, n ( %)	4620 (18.2)	248 (14.6)	944 (16.7)	2124 (17.8)	1304 (21.2)	<0.001	1616 (16.1)	2147 (18.5)	692 (23.3)	<0.001
Anti-hypertensive Agents, n (%)										
ACE inhibitor, n (%)	2121 (8.3)	172 (10.1)	479 (8.5)	959 (8.0)	511 (8.3)	0.032	794 (7.9)	992 (8.6)	252 (8.5)	0.167
angiotensin II receptor antagonist, n (%)	4084 (16.0)	262 (15.5)	885 (15.6)	1861 (15.6)	1076 (17.5)	0.006	1272 (12.7)	2059 (17.8)	586 (19.8)	<0.001
Calcium channel blockers, n (%)	6202 (24.4)	451 (26.6)	1497 (26.4)	2847 (23.9)	1407 (22.9)	<0.001	2356 (23.5)	2960 (25.5)	662 (22.3)	<0.001
Beta-blocker	2361 (9.3)	143 (8.4)	518 (9.1)	1097 (9.2)	603 (9.8)	0.306	744 (7.4)	1181 (10.2)	316 (10.7)	<0.001
Lipid Lowering Agents, n (%)										
Statins, n (%)	5054 (19.9)	301 (17.8)	1045 (18.4)	2251 (18.9)	1457 (23.7)	<0.001	1662 (16.6)	2425 (20.9)	738 (24.9)	<0.001
Aspirin, n (%)	4693 (18.4)	292 (17.2)	1013 (17.9)	2169 (18.2)	1219 (19.8)	0.012	1615 (16.1)	2334 (20.1)	584 (19.7)	<0.001

^a^865 patients did not have household income record. *OHD* oral hypoglycemic drug; *ACE* angiotensin-converting enzyme

**Fig. 1:**
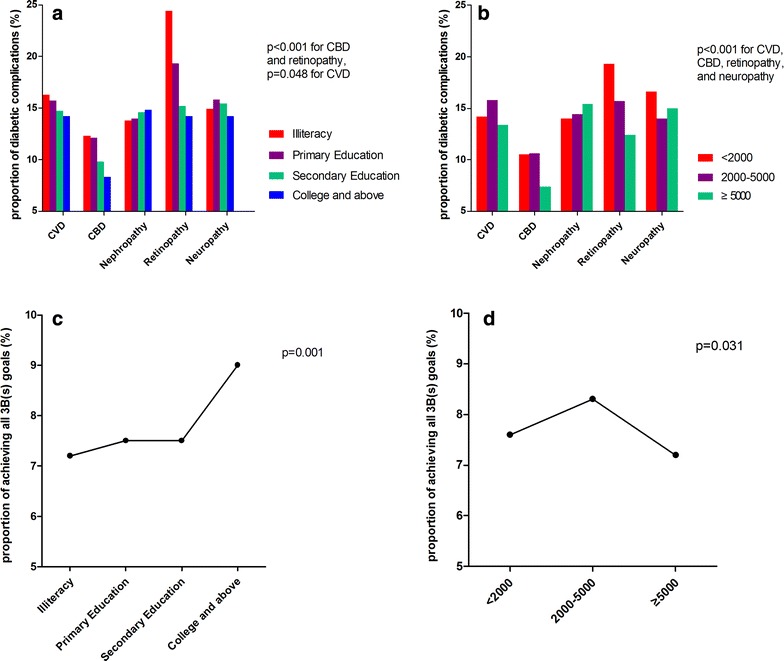
Diabetes complications and 3B control based on different education and income levels. **a** Diabetes complications among study participants by education level and **b** by income level. **c** Achievement of 3B control among study participants by education level and **d** by income level. *CVD* cardiovascular disease, *CBD* cerebrovascular disease

The patterns of 3B (blood glucose, blood pressure, and blood lipids) control and diabetes complications are presented in Table 3 and Fig. 1. Among the education groups, HbA1c values did not differ significantly in males (*p* = 0.169), but decreased with increasing education level in females (*p* < 0.05). The values of SBP were higher in patients with less education, whereas among the education groups, patients with higher education level had relatively higher TC, TG, and LDL values. Diabetes complications were more prevalent in patients with a lower education level [*p* < 0.001 for cerebrovascular disease (CBD) and retinopathy, *p* = 0.048 for cardiovascular disease (CVD); Fig. 1a). The percentage of patients who achieved all 3B goals was 9.0 % in the college and above group, which was higher than the percentages in other groups (*p* = 0.001, Fig. 1c). Similarly, the values of DBP and fasting blood glucose decreased with increasing income. However, patients with the highest income had the highest HbA1c level (*p* < 0.001). The incidences of complications decreased with increasing income level (*p* < 0.001 for CVD, CBD, retinopathy, and neuropathy; Fig. 1b). However, the percentage of patients who achieved all 3B goals was only 7.2 % in the highest income group, which was significantly lower that in than other two groups (*p* = 0.031, Fig. 1d).

**Table 3:** The control of 3B(s) in male or female patients shown by education and income levels

Variables	All patients	Education					Household net income^a^			
		Illiteracy	Primary education	Secondary education	College and above	*p*	<2000	2000–5000	≥5000	*p*
Male, n	11,955	277	1836	5738	4104		3941	5796	1804	
SBP (mmHg), mean ± SD	132.5 ± 15.48	136.3 ± 16.23	134.4 ± 15.87	132.6 ± 15.90	131.2 ± 14.51	<0.001	133.5 ± 16.15	132.0 ± 15.10	131.2 ± 14.57	<0.001
DBP (mmHg), mean ± SD	79.4 ± 9.03	79.2 ± 9.30	78.6 ± 9.37	79.7 ± 9.08	79.5 ± 8.75	<0.001	79.7 ± 9.21	79.3 ± 8.92	78.9 ± 8.70	<0.001
HbA1c (%), mean ± SD	7.7 ± 2.07	7.6 ± 2.16	7.8 ± 2.20	7.8 ± 2.10	7.7 ± 1.97	0.169	7.7 ± 2.09	7.7 ± 2.07	7.8 ± 2.07	0.015
FBG (mmol/L), mean ± SD	8.5 ± 3.45	8.4 ± 4.25	8.6 ± 3.81	8.6 ± 3.42	8.3 ± 3.26	0.009	8.6 ± 3.50	8.5 ± 3.50	8.4 ± 3.28	0.040
TC (mmol/L), mean ± SD	4.8 ± 1.55	4.7 ± 1.23	4.7 ± 1.24	4.8 ± 1.77	4.8 ± 1.35	0.004	4.7 ± 1.45	4.8 ± 1.61	4.8 ± 1.64	0.035
TG (mmol/L), median (IQR)	1.5 (1.0–2.3)	1.3 (1.0–1.9)	1.4 (1.0–2.0)	1.5 (1.0–2.3)	1.6 (1.1–2.4)	<0.001	1.5 (1.0–2.2)	1.5 (1.0–2.3)	1.5 (1.1–2.3)	0.521
LDL (mmol/L), mean ± SD	2.7 ± 0.88	2.6 ± 0.90	2.7 ± 0.89	2.7 ± 0.87	2.8 ± 0.89	<0.001	2.7 ± 0.87	2.8 ± 0.88	2.8 ± 0.93	0.040
HDL (mmol/L), mean ± SD	1.2 ± 0.47	1.2 ± 0.38	1.2 ± 0.45	1.2 ± 0.46	1.2 ± 0.48	<0.001	1.2 ± 0.45	1.2 ± 0.49	1.2 ± 0.41	<0.001
Female, n	13,499	1418	3831	6198	2025		6098	5790	1160	
SBP (mmHg), mean ± SD	133.4 ± 15.95	135.3 ± 16.84	135.5 ± 16.27	132.4 ± 15.69	131.3 ± 14.93	<0.001	133.8 ± 16.47	132.8 ± 15.20	133.2 ± 15.90	<0.001
DBP (mmHg), mean ± SD	78.2 ± 8.87	77.8 ± 8.98	78.4 ± 9.04	78.3 ± 8.82	77.6 ± 8.59	0.001	78.7 ± 8.96	77.9 ± 8.69	76.7 ± 8.47	<0.001
HbA1c (%), mean ± SD	7.5 ± 1.98	7.6 ± 2.12	7.6 ± 2.02	7.5 ± 1.96	7.4 ± 1.83	0.012	7.5 ± 2.03	7.5 ± 1.93	7.7 ± 1.92	0.008
FBG (mmol/L), mean ± SD	8.3 ± 3.31	8.6 ± 3.65	8.5 ± 3.43	8.3 ± 3.24	8.0 ± 2.99	<0.001	8.5 ± 3.49	8.2 ± 3.15	8.1 ± 3.12	<0.001
TC (mmol/L), mean ± SD	5.2 ± 1.33	5.1 ± 1.22	5.1 ± 1.33	5.2 ± 1.38	5.2 ± 1.20	0.014	5.1 ± 1.25	5.2 ± 1.41	5.1 ± 1.25	0.040
TG (mmol/L), Median (IQR)	1.6 (1.1–2.3)	1.5 (1.1–2.3)	1.6 (1.2–2.3)	1.6 (1.1–2.3)	1.6 (1.1–2.2)	0.142	1.6 (1.2–2.3)	1.6 (1.1–2.3)	1.5 (1.1–2.2)	0.248
LDL (mmol/L), mean ± SD	2.9 ± 0.93	2.9 ± 0.93	2.9 ± 0.94	2.9 ± 0.92	2.9 ± 0.92	0.002	2.9 ± 0.92	2.9 ± 0.92	2.9 ± 0.96	0.107
HDL (mmol/L), mean ± SD	1.4 ± 0.56	1.3 ± 0.47	1.4 ± 0.51	1.4 ± 0.50	1.4 ± 0.84	0.049	1.4 ± 0.46	1.4 ± 0.68	1.3 ± 0.45	<0.001

414 male patients and 451 female patients without household income record. FBG: fasting blood glucose; *SBP* systolic blood pressure, *DBP* diastolic blood pressure, *TC* total cholesterol; *TG* triglycerides, *LDL* low-density lipoprotein, *HDL* high-density lipoprotein

Continuous variables were analyzed using the Student’s t test and ANOVA. Categorical variables were analyzed using the Chi squared test and Fisher’s exact test

Bivariate correlation analyses showed that a high education level was strongly correlated with both achievement of HbA1c target (OR 1.38, *p* < 0.001) and achievement of all 3B goals (OR 1.30, *p* = 0.001) after adjustment for age, gender, BMI, smoking, alcohol consumption, exercise, and diabetes duration (Table 4). High income was correlated with achievement of BP target (OR 1.16, *p* = 0.001) and poor glycemic control (OR 0.90, *p* = 0.021), whereas medium income was correlated with achievement of TC target (OR 1.20, *p* = 0.015) after adjustment for age, gender, BMI, smoking, alcohol consumption, exercise, and diabetes duration (Table 5). The associations between diabetes complications and SES were also explored by logistic regression analysis, which indicated that a high education level was correlated with less chance of developing CVD, CBD, or retinopathy (OR = 0.79 and p = 0.003, OR 0.68 and p < 0.001, and OR 0.58 and p < 0.001, respectively), and a high household net income level was correlated with less chance of developing CBD or retinopathy (OR 0.76 and p = 0.001 and OR 0.65 and p < 0.001, respectively), as shown in Additional file 1: Table S1.

**Table 4:** The odds ratio of education for control of 3B (s)

Dependent variables	Independent variables	Unadjusted		Model 1		Model 2	
		OR (95 %CI)	*p*	OR (95 %CI)	*p*	OR (95 %CI)	*p*
HbA1c <7.0 %	College and above	1.09 (0.97, 1.21)	0.153	1.40 (1.24, 1.57)	<.001	1.38 (1.22, 1.56)	<.001
	Secondary Education	1.01 (0.90, 1.13)	0.882	1.09 (0.98, 1.22)	0.114	1.07 (0.96, 1.20)	0.228
	Primary Education	1.00 (0.91, 1.11)	0.935	1.23 (1.11, 1.37)	<.001	1.23 (1.10, 1.37)	<.001
	Illiteracy	1		1		1	
BP <140/80 mmHg	College and above	1.03 (0.92, 1.15)	0.624	1.09 (0.97, 1.23)	0.146	1.07 (0.95, 1.21)	0.280
	Secondary Education	1.01 (0.90, 1.13)	0.898	1.03 (0.92, 1.15)	0.639	1.02 (0.91, 1.14)	0.782
	Primary Education	1 (0.90, 1.11)	0.996	1.04 (0.93, 1.16)	0.501	1.02 (0.92, 1.14)	0.703
	Illiteracy	1		1		1	
TC <4.5 mmol/L	College and above	1.15 (1.03, 1.30)	0.017	1.02 (0.90, 1.150)	0.816	1.02 (0.90, 1.15)	0.799
	Secondary Education	1.12 (0.10, 1.25)	0.062	1.07 (0.95, 1.20)	0.275	1.07 (0.95, 1.20)	0.262
	Primary Education	1.09 (0.98, 1.21)	0.125	1.04 (0.93, 1.17)	0.495	1.04 (0.92, 1.16)	0.547
	Illiteracy	1		1		1	
BP 140/80 mmHg, HbA1c <7.0 % and TC <4.5 mmol/L	College and above	1.28 (1.10, 1.49)	0.002	1.32 (1.12, 1.54)	<.001	1.30 (1.11, 1.53)	0.001
	Secondary Education	1.12 (0.96, 1.30)	0.141	1.12 (0.97, 1.31)	0.134	1.11 (0.96, 1.30)	0.165
	Primary Education	1.14 (0.99, 1.32)	0.066	1.23 (1.06, 1.42)	0.007	1.22 (1.05, 1.41)	0.001
	Illiteracy	1		1		1	

*Model 1* adjusted for age and gender

*Model 2* adjusted for age, gender, BMI, smoking, alcohol consumptions, exercise and diabetes duration

Data were analyzed using a multivariable logistic regression analysis

**Table 5:** The odds ratio of household net income for control of 3B (s)

Dependent variables	Independent variables	Unadjusted		Model 1		Model 2	
		OR (95 %CI)	*p*	OR (95 %CI)	*p*	OR (95 %CI)	*p*
HbA1c <7.0 %	≥5000	0.83 (0.76, 0.91)	<.001	0.86 (0.78, 0.93)	<.001	0.90 (0.83, 0.98)	0.021
	2000–5000	0.89 (0.77, 1.02)	0.088	0.89 (0.78, 1.03)	0.120	0.93 (0.80, 1.07)	0.302
	<2000	1		1		1	
BP <140/80 mmHg	≥5000	1.13 (1.04, 1.23)	0.006	1.15 (1.05, 1.25)	0.002	1.16 (1.07, 1.27)	<.001
	2000–5000	0.90 (0.78, 1.04)	0.161	0.91 (0.79, 1.05)	0.193	0.91 (0.79, 1.05)	0.197
	<2000	1		1		1	
TC <4.5 mmol/L	≥5000	1.11 (1.02, 1.21)	0.018	1.06 (0.97, 1.16)	0.199	1.06 (0.97, 1.16)	0.190
	2000–5000	1.23 (1.06, 1.42)	0.005	1.20 (1.04, 1.38)	0.015	1.20 (1.04, 1.38)	0.015
	<2000	1		1		1	
BP <140/80 mmHg, HbA1c <7.0 % and TC <4.5 mmol/L	≥5000	0.95 (0.85, 1.07)	0.389	0.93 (0.83, 1.04)	0.120	0.96 (0.86, 1.08)	0.501
	2000–5000	1.00 (0.83, 1.20)	0.991	0.98 (0.82, 1.18)	0.834	1.01 (0.84, 1.21)	0.929
	<2000	1		1		1	

*Model 1* adjusted for age and gender

*Model 2* adjusted for age, gender, BMI, smoking, alcohol consumptions, exercise and diabetes duration

Data were analyzed using a multivariable logistic regression analysis

## Discussion

To the best of our knowledge, this is the first nationwide study investigating 3B control and diabetes complications in relation to individual SES level in a Chinese population. Our study showed that the most educated patients showed the best achievement of HbA1c target and all 3B goals, and vice versa, the least educated patients had the highest incidences of CVD, CBD, and retinopathy. The patients with highest income showed the best achievement of BP target but worst achievement of HbA1c target. The lowest income patients had the highest incidences of retinopathy and neuropathy.

Education is the most commonly used measure of SES in epidemiological studies. Those with the lowest educational attainment have been reported to exhibit the highest prevalence of CVD [18]. Another study reported that the mean values of HbA1c and TC are higher in primary-educated type 1 diabetes patients than in their college-educated counterparts [1]. Bachmann et al. [3] found that the least educated patients are more likely than the most educated patients to have retinopathy, heart disease, and higher HbA1c levels. However, there seems to be no association between education level and glycemic control [5]. Moreover, educational level is a strong predictor of mortality among adults with diabetes [6]. Recent clinical studies revealed that low SES is associated with a higher prevalence of diabetes and its complications, worse outcomes, and worse quality of care, suggesting that tailored interventions for socially disadvantaged patients can have positive effects on diabetes care [19–21]. In our study, higher education was suggestive of a lower risk of developing diabetes complications, such as CVD, CBD, and retinopathy, and the correlations were strong for both achievement of HbA1c target and achievement of all 3B goals, although no correlation between SES and the achievement of BP or TC was found. Further studies in patients with high levels of education are needed to explain these findings according to multiply aspects such as lifestyle. Unexpectedly, patients with secondary education did not exhibit an advantage in achieving all 3B goals over patients with primary education.

Poverty is associated with a higher incidence of diabetes in Asian countries [14, 22, 23], probably because income enables individuals to purchase various goods and services to improve health care. It has been reported that poorer individuals have higher HbA1c levels than those with higher income. Bachmann et al. [3] found that the adjusted odds of retinopathy are four times higher in the lowest earning patients, compared with the highest earning patients. However, income was found to not be associated with blood glucose, cholesterol, or blood pressure [3]. In the present study, patients with high income had less chance of developing diabetes complications, such as CVD, CBD, and retinopathy, and high income was found to be correlated with achievement of the BP target. Interestingly, medium income was correlated with achievement of the TC target. However, higher income patients in China tended to have worse HbA1c levels, and only 7.2 % of patients with the highest income achieved all 3B goals, a proportion lower than those in the other two groups. Consistent with our findings, another clinical study in China indicated that higher income is related to a greater prevalence of type 2 diabetes, and high BMI was responsible for this association [24]. The possible reasons for bad glycemic and 3B control in the high income population involved a more diversified diet and frequent dinner party attendance due to the wider social network of individuals in this group. Notably, the prevalence of undiagnosed diabetes was 8.1 % (95 % CI, 7.9–8.3 %) in the Chinese population, and also sometimes individuals were diagnosed with diabetes very late. These undiagnosed patients need special efforts to control their cardiovascular risks, especially in those with lower SES [25].

Our study enrolled a nationally representative sample of 25,454 patients from 104 hospitals across China, providing new evidence from the real world setting. However, it still has several limitations that should be noted. First, it was a cross-sectional study that did not assess long-term outcomes. It is difficult to determine causal relationships between SES and health outcomes. Further studies are needed to explore the mediators through which SES influences health outcomes in Chinese patients with type 2 diabetes. Second, because the parameters (blood lipids, HbA1c, etc.) were not measured in a central laboratory and self-reported SES measures were used, systematic bias due to lack of standardized assessment may exist.

## Conclusions

Overall, lower levels of education and income are associated with worse metabolic control and more diabetes complications, i.e., retinopathy and cerebrovascular diseases, in China. Based on individual features of SES, diabetes patients are encouraged to enhance their disease management with the help from social support or medical staff in order to reduce risks of complications, facilitate achievement of metabolic control, and thus improve health outcomes.

## Additional file

10.1186/s12933-016-0376-7 Association between Diabetic Complications and SES by logistic regression.

## Abbreviations

SES: socioeconomic status
3Bs: blood glucose, blood pressure, blood cholesterol
OR: adjusted odds ratio
HbA1c: hemoglobin A1c
3B STUDY: Nationwide Assessment of Cardiovascular Risk Factors: blood glucose, blood pressure, and blood lipid in Chinese Patients with Type 2 Diabetes
CCMR: China Cardiometabolic Registries
SBP and DBP: systolic and diastolic blood pressure
TC: total serum cholesterol
BP: blood pressure
FBG: fasting blood glucose
LDL: low-density lipoprotein
HDL: high-density lipoprotein
OHD: oral hypoglycemic drugs
TZDs: thiazolidinediones
ACE: angiotensin-converting enzyme
CBD: cerebrovascular diseases
CVD: cardiovascular diseases
