# Supplementary material for: Association between socioeconomic status and metabolic control and diabetes complications: a cross-sectional nationwide study in Chinese adults with type 2 diabetes mellitus
Source: Cardiovasc Diabetol. 2016 Apr 5;15:61. doi: 10.1186/s12933-016-0376-7 (PMC4822246; doi:10.1186/s12933-016-0376-7)
Supplement: Supplementary file 1 — 10.1186/s12933-016-0376-7 Association between Diabetic Complications and SES by logistic regression. [file 12933_2016_376_MOESM1_ESM.docx]

Supplement Table 1 Association between Diabetic Complications and SES by logistic regression

|  | CVD | | CBD | | Nephropathy | | Retinopathy | | Neuropathy | |
| --- | --- | --- | --- | --- | --- | --- | --- | --- | --- | --- |
|  | OR(95%CI) | *p* | OR(95%CI) | *p* | OR(95%CI) | *p* | OR(95%CI) | *p* | OR(95%CI) | *p* |
| Household Net Income |  |  |  |  |  |  |  |  |  |  |
| <2000 (reference) | 1 |  | 1 |  | 1 |  | 1 |  | 1 |  |
| 2000-5000 | 1.19(1.10-1.28) | <0.001 | 1.10(1.01-1.21) | 0.037 | 1.01(0.94-1.10) | 0.719 | 0.84(0.78-0.91) | <0.001 | 0.82(0.76-0.89) | <0.001 |
| ≥5000 | 0.99(0.87-1.12) | 0.852 | 0.76(0.65-0.89) | 0.001 | 1.09(0.97-1.23) | 0.157 | 0.65(0.57-0.73) | <0.001 | 0.90(0.80-1.01) | 0.073 |
| Education |  |  |  |  |  |  |  |  |  |  |
| Illiteracy (reference) | 1 |  | 1 |  | 1 |  | 1 |  | 1 |  |
| Primary Education | 0.92(0.79-1.07) | 0.274 | 1.02(0.86-1.21) | 0.800 | 1.06(0.90-1.25) | 0.483 | 0.76(0.67-0.87) | <0.001 | 1.14(0.97-1.33) | 0.106 |
| Secondary Education | 0.84(0.72-0.97) | 0.015 | 0.78(0.66-0.92) | 0.004 | 1.11(0.96-1.31) | 0.158 | 0.58(0.51-0.66) | <0.001 | 1.14(0.98-1.32) | 0.093 |
| College and above | 0.79(0.67-0.92) | 0.003 | 0.68(0.57-0.82) | <0.001 | 1.12(0.96-1.34) | 0.153 | 0.58(0.51-0.67) | <0.001 | 1.07(0.91-1.25) | 0.416 |

CVD: Cardiovascular Disease; CBD: Cerebrovascular Disease. Data were analyzed using a multivariable logistic regression analysis.
